# Efficacy of a Smartphone-Based Virtual Patient in Increasing Clinical Competence Among Primary Health Care Providers: Protocol for a Cluster Randomized Controlled Trial

**DOI:** 10.2196/106849

**Published:** 2026-11-06

**Authors:** Xiaohui Wang, Kaixuan Zhang, Jie Du, Yiyuan Cai, Jin Cao, Yaolong Chen, Liwen Ding, Yuan Fang, Wenjie Gong, Wenjun He, Jinghua Li, Huijuan Liang, Jing Liao, Yun Lu, Baibing Mi, Jay Pan, Ruixin Wang, Dong Roman Xu, Nan Zhang, Qing Zhao, Jifang Zhou, Zhongliang Zhou, Zixin Wang

**Affiliations:** 1 Department of Health Policy and Management, School of Public Health, Lanzhou University, Lanzhou, Gansu, China; 2 Department of Psychological Medicine, Peking Union Medical College Hospital, Chinese Academy of Medical Sciences & Peking Union Medical College, Beijing, China; 3 Department of Epidemiology and Health Statistics, School of Public Health, Guizhou Medical University, Guiyang, Guizhou, China; 4 Institute of Health Data Science / Evidence-Based Medicine Center, Lanzhou University, Lanzhou, China; 5 Centre for Health Behaviours Research, JC School of Public Health and Primary Care, The Chinese University of Hong Kong, Room 508, Prince of Wales Hospital, Hong Kong, China (Hong Kong), 852 22528474; 6 HER Team and Department of Maternal and Child Health, Xiangya School of Public Health, Central South University, Changsha, Hunan, China; 7 School of Health Management, Southern Medical University, Guangzhou, Guangdong, China; 8 Department of Public Health and Medicinal Administration, Faculty of Health Sciences, University of Macau, Macau, Macao; 9 School of Health Management, Inner Mongolia Medical University, Hohhot, Inner Mongolia, China; 10 Department of Medical Statistics and Epidemiology, School of Public Health, Sun Yat-sen University, Guangzhou, Guangdong, China; 11 Department of Preventive Medicine, Maternal and Child Health, School of Public Health, School of Public Health, Guizhou Medical University, Guizhou, China; 12 Department of Epidemiology and Biostatistics, School of Public Health, Xi’an Jiaotong University Health Science Center, Xi'an, Shanxi, China; 13 HEOA Group, West China School of Public Health and West China Fourth Hospital, Sichuan University, Chengdu, Sichuan, China; 14 Department of Health Economics, School of Public Health, Fudan University, Shanghai, China; 15 Southern Medical University Institute for Global Health (SIGHT), Dermatology Hospital of Southern Medical University, Guangzhou, China; 16 Acacia Lab for Implementation Science, School of Health Management and Center for World Health Organization Studies, Southern Medical University, Guangzhou, China; 17 School of International Business, China Pharmaceutical University, Nanjing, Jiangsu, China; 18 School of Public Policy and Administration, Xi'an Jiaotong University, Xi’an, Shaanxi, China

**Keywords:** smartphone-based virtual patients, diagnosis and treatment competence, primary health care physicians, cluster randomized controlled trial, resource-limited settings

## Abstract

**Background:**

Virtual patients (VPs) are considered cost-effective and convenient tools for clinical training. The research team developed a smartphone-based VP tailored to the needs of primary health care physicians in rural China. The VP demonstrated high levels of feasibility and acceptability in this group in a previous study.

**Objective:**

The proposed cluster randomized controlled trial (RCT) will compare the efficacy of clinical training delivered via a smartphone-based VP in increasing diagnosis and treatment competence among primary health care providers in rural China as compared to a waitlist control group.

**Methods:**

This is a partially blinded (outcome assessors and data analysts) and parallel-group cluster RCT conducted in Lintao county, China. All 18 township health centers in Lintao will be randomized evenly into either the intervention or the waitlist control group. The target sample size is 150 physicians per group. Physicians in the intervention group will receive clinical training delivered via the smartphone-based VP. They will be required to complete the diagnosis and treatment plan of 2 conditions randomly assigned by the VP within a 1-month intervention period. The VP will provide detailed feedback on their procedures. The intervention group can also access 9 other conditions in the VP and receive detailed feedback during the intervention period. Participants in the control group will not have access to any clinical training delivered via the VP during the 1-month intervention period. They will gain full access to the VP system after completion of the outcome assessment at T1 (1 month after baseline [T0]). The primary outcome is the score representing overall diagnosis and treatment competence. To measure this outcome, participants will be invited to complete the diagnosis and treatment plan of a condition randomly assigned by the smartphone-based VP at both T0 and T1. Intention-to-treat analysis will be performed.

**Results:**

Study recruitment started on August 1, 2026. By August 15, 2026, a total of 18 township health centers had been randomly assigned to either the intervention (9 centers) or control (9 centers) group. A total of 216 physicians from these centers completed baseline assessment; 56% (121/216) were randomly assigned to the intervention group, and 44% (95/216) were assigned to the control group. Recruitment will be completed by the end of November 2026. The follow-up assessment at T1 will start in September 2026. Data collection is expected to be concluded in January 2027.

**Conclusions:**

The findings will extend the application of smartphone-based VPs in medical training. If the smartphone-based VP is proven effective in increasing diagnosis and treatment competence among primary health care physicians in rural China, it will provide a feasible and effective alternative training tool to address the existing service gaps.

## Introduction

### Background

Universal health coverage is the primary goal for health system development across countries [1]. Primary health care services play important roles in achieving universal health coverage [2]. Increasing service coverage alone may not be sufficient to improve health outcomes [3]. It is also necessary to improve the quality of primary health care services [3]. Worldwide, low-quality health care services result in over 5 million deaths annually, including 653,000 deaths in China [4]. Valid, low-cost, and easy-to-implement training tools may be useful to improve the quality of primary health care services, especially in resource-limited settings [5].

Virtual patients (VPs) are sophisticated educational simulations designed to replicate authentic clinical scenarios, which enable health care trainees to practice skills in a safe and controlled environment without risk to real patients [6]. VPs are used to address several learning outcomes in the education of health professionals [7]. A common feature of VPs is that they can simulate patients representing different conditions and allow the user to act as a medical professional [8]. Through the interactions with the VP, the user can obtain medical history, conduct physical examinations, order laboratory testing, and provide a diagnosis and management plan [8]. In addition, the VP system is able to provide detailed feedback on users’ diagnosis and treatment procedures [8]. A strength of VPs is that they ensure that trainees encounter patients representing a wide range of conditions, which might not always be possible during clinical rotation [9,10]. VPs are considered cost-effective and convenient tools for clinical training [11,12]. Published systematic reviews support the effectiveness of using VPs to improve medical students’ clinical reasoning skills [13]. Meta-analyses of randomized controlled trials (RCTs) have shown a positive pooled overall effect of VPs compared to other medical educational methods [14]. Positive effects have been found when VPs were used both as a stand-alone or additive resource [14]. However, most of the previous RCTs have targeted medical students. There is a dearth of RCTs evaluating the efficacy of VPs in improving diagnosis and treatment competence among primary health care providers.

VPs can be implemented on mobile devices (eg, smartphones), which provides better access to training resources for trainees [7,11,12,15]. A smartphone-based VP was developed in China by a joint working group led by the Global Health Research Centre of the Southern Medical University of China [11,12]. This smartphone-based VP was evaluated by a panel of experts. The conditions simulated by the VP and the assessment tool for user performance demonstrated good content and face validity [11,12]. A study was conducted by the research team to investigate the feasibility and acceptability of the smartphone VP among physicians in township health centers or village clinics in rural China. The feasibility was high as 146 out of 158 participants (92.4%) completed both conditions assigned by the VP, while 12 (7.6%) completed one condition. Overall, 79.9% to 83.2% of the participants agreed with the items reflecting utility, and 79.9% to 85.9% were satisfied with the VP system.

### Objectives

The proposed cluster RCT will compare the efficacy of clinical training delivered via a smartphone-based VP in increasing diagnosis and treatment competence among primary health care providers in rural China as compared to a waitlist control group. The diagnosis and treatment competence will be measured via the smartphone-based VP using a validated checklist both at baseline (T0) and after completion of the 1-month intervention period (T1). Our hypothesis is that participants in the intervention group will have better diagnosis and treatment competence compared to those in the waitlist control group at T1.

## Methods

### Study Design

This is a partially blinded (outcome assessors and data analysts) and parallel-group cluster RCT conducted in Lintao county, China. During the funding application, we intended to select township health centers in pairs based on their geographic location, size, and number of eligible physicians working in the center. During the program implementation, we were not able to use the proposed matched-pair design for the following reasons: (1) only 1 township center in some regions consented to participate in this study and (2) substantial differences in size or number of eligible physicians between consented township health centers within the same region. Therefore, we changed our design to a parallel-group cluster RCT. There was no change in the intervention, outcomes, or analysis after funding. The aforementioned changes are reflected in the current ethics-approved protocol and trial registration. Lintao is an underdeveloped, multiethnic dwelling county in Gansu province located in western China, with a population size of 0.5 million. All 18 township health centers in Lintao will be randomized evenly into either the intervention or the waitlist control group. Physicians in the township health centers that are allocated to the intervention group will receive clinical training delivered via the smartphone-based VP. These physicians will be required to complete the diagnosis and treatment plan of 2 conditions randomly assigned by the VP. The VP will provide detailed feedback on their procedures. Participants in the intervention group can also access 9 other conditions in the VP and receive detailed feedback, and they are free to spend as much time as they want to on such functions. Participants in the township health centers that are allocated to the control group will not have access to any clinical training delivered via the VP before T1 and will gain access to such functions at T1. This study was prospectively registered in the Chinese Clinical Trial Registry (reference number ChiCTR2600127364).

### Participants and Recruitment

Participants will be physicians employed by township health centers. Physicians who plan to leave their current position or health center in the next 6 months or without access to smartphones will be excluded. We have approached all 18 township health centers in Lintao county, and all of them provided consent to join this study. All eligible physicians working in the participating township health centers will be invited to join the study. The research team will visit the selected health centers in person, assess the eligibility of prospective physicians, brief them about the study, and invite them to participate. Prospective participants will be briefed that their interaction with the VP will be encrypted and will not be used by the VP developer or any other parties. Only the principal investigator of this study has access to the data. The VP-human interaction history will be removed from the VP server once the project has ended. The research team will also guarantee that the participants have the right to quit at any time and refusal or withdrawal will have no consequences. Participants can approach the research team for inquiries via telephone or instant messaging applications during the study period. According to records, there are 408 eligible physicians in these centers. On the basis of the response rate in the aforementioned previous feasibility study (ie, 93.5%), it is feasible to meet our target sample size (N=300).

### Sample Size Planning

The sample size is calculated using the following formula: $n=\frac{2{\left(Z_{\alpha /2}\;+\;Z_{\beta }\right)}^{2}\sigma^{2}\times Design\;Effect\;\left(DE\right)}{\delta^{2}}$. In this formula, σ represents the SD of the primary outcome (ie, score representing overall diagnosis and treatment competence), which was 10 in our feasibility study. δ represents the minimal between-group difference in the primary outcome with the smallest meaningful difference and is set to 5 in this study. The design effect (DE) is calculated using the following formula: DE = 1 + (average number of participants per cluster − 1) × intracluster correlation coefficient. In this study, the average number of participants per cluster is 22, and the intracluster correlation coefficient is set at 0.05. On the basis of the aforementioned formula and parameters, a sample size of 135 per group (270 in total) is needed (α=.05; power=0.80). Assuming the dropout rate at T1 is 10%, the final sample size would be 150 per group (300 in total).

### Ethical Considerations

Ethics approval was obtained from the ethics committee of the School of Public Health, Lanzhou University (reference IRB18091501; approval date: September 15, 2018). The procedures of this study were in accordance with the ethical standards of the aforementioned responsible committees and with the World Medical Association Declaration of Helsinki. Written informed consent will be obtained from the participants prior to the start of this study.

### Development of the Smartphone-Based VP

#### Overview

Details of the development and validation process of the VP are described in published papers [11,16]. A panel consisting of experts in evidence-based medicine, experienced physicians, and physicians working in primary health care settings in rural China from the relevant specialties was formed. The development procedures were conducted in the following steps.

#### Step 1: Selecting Conditions

The panel selected conditions that could represent the patients of primary health care settings in rural China based on a comprehensive review of literature and medical records and clinical experiences from frontline primary health care physicians. The selection was based on (1) high frequency of clinical encounters in primary health care settings in rural areas, (2) significance of the disease burden, and (3) representation of the major working areas of primary health care settings in rural areas. Multiple panel discussions were held to reach consensus, and 11 conditions were selected: low back pain, cold, pediatric diarrhea, stress urinary incontinence, postpartum depression, hypertension, diabetes mellitus, asthma, angina pectoris, gastritis, and migraine.

#### Step 2: Selecting Standard Clinical Procedures and Quality Assessment Tools

Led by experts in evidence-based medicine, a systematic review was performed to retrieve the standardized procedures for diagnosis and management of the aforementioned conditions and quality assessment of these clinical procedures in primary health care settings. Relevant literature was systematically retrieved and evaluated for quality using standardized tools such as the Appraisal of Guidelines for Research and Evaluation II for guidelines [17], A Measurement Tool to Assess Systematic Reviews for systematic reviews [18], or Quality Assessment of Diagnostic Accuracy Studies–2 for diagnostic studies [19]. Through panel discussion, consensus was reached for the standard clinical procedures for each condition and the tools to assess the quality of these procedures.

#### Step 3: Developing a Diagnosis and Treatment Checklist for Each Selected Condition

On the basis of the findings of the previous steps, a preliminary checklist for each selected condition was developed by the panel. The checklist was structured into 5 domains, including a patient’s background information and medical history, physical examination results, laboratory and imaging reports, diagnosis, and management and treatment plans to simulate real-life clinical scenarios. Such checklists were identical to unannounced standardized patients simulated by volunteers.

#### Step 4: Finalizing the Checklists

Three rounds of expert consultation were conducted to achieve consensus among clinical experts on the items of the diagnosis and treatment checklists. The panel modified the checklists based on the feedback obtained from the expert consultation and pilot-tested the checklists in medical institutions to ensure that the items were unambiguous and the scenario was clinically realistic.

#### Step 5: Implementing the Checklists as a Smartphone-Based VP

The finalized checklists for the selected conditions were implemented on CureFun, an existing smartphone-based training platform using VPs [11,12]. The smartphone-based VP could present interactive clinical scenarios and automatically record users’ diagnosis and treatment pathways and grade them based on the aforementioned assessment tools.

### Baseline Survey and Randomization

At baseline, a self-administered web-based survey was used to collect users’ background characteristics, including age, gender, ethnicity, educational level, training background, and years of working experience. After completion of the web-based baseline survey, participants in both groups will be granted access to the smartphone-based VP. The app plays a demonstration video at the first use, and the users can watch it repeatedly. Participants will be invited to complete the diagnosis and treatment plan of a condition randomly assigned by the smartphone-based VP to document the primary outcome at T0. Participating township health centers will be allocated randomly to 1 of the 2 study groups based on a computer-generated list. Block randomization (block size of 4) will be used to generate group assignment. The list will be stored at the Institute of Social Medicine and Health Management, School of Public Health, Lanzhou University. Group allocation will be performed based on the randomization list. The list will be concealed from individuals involved in the recruitment process and in communication with township health centers.

### Intervention Group

In addition to the outcome assessments at T0 and T1, physicians in the intervention group will have full access to the VP system during the intervention period. They will be required to complete the diagnosis and treatment plan of 2 conditions randomly assigned by the VP. In the interface of the VP, users can interact with the VP by selecting items within the modules of history taking, physical examination, auxiliary tests, and screening scales to gather clinical information. They can then make clinical decisions through the diagnosis and management modules. The VP will provide detailed feedback on their procedures. Participants can also access 9 other conditions in the VP and receive detailed feedback, and they are free to spend as much time as they want to on such functions.

### Control Group

The control group will not have access to any functions of the VP other than the assessment at T0 and T1. They will gain full access to the VP system after completion of the outcome assessment at T1.

### Outcome Measurements

The primary outcome of this study is the score representing overall diagnosis and treatment competence. To measure this outcome, participants will be invited to complete the diagnosis and treatment plan of a condition randomly assigned by the smartphone-based VP. The VP system will rate the performance of the participants using a validated checklist in accordance with the National Academy of Medicine quality assessment framework. The checklist contains five domains: (1) how close the clinical pathway implemented by the user matches the evidence-based pathway recommended by the clinical practice guidelines (0-30 marks), (2) accuracy of diagnosis (accurate primary diagnosis=22 marks; inaccurate primary diagnosis=0 marks), (3) collection of clinical information (0-31 marks), (4) appropriate treatment and management plan (0-12 marks), and (5) whether the cost of management exceeds the average level (yes=0 marks; no=5 marks). The VP system will sum the scores of all 5 domains to generate an overall score. An overall score of less than 60 marks indicates insufficient overall diagnosis and treatment competence, while a score of 60 marks or higher indicates adequate competence. If users obtain a score of 80 or above, they will be considered to have excellent competence. Meanwhile, the VP system will also monitor whether users have violated clinical safety. In case a clear violation is recorded, users will receive 0 marks for the overall competence.

### Statistical Analysis

Intention-to-treat analysis will be used. Multiple imputation (5 times) will be used to replace the missing outcomes at T1. Assuming the data are missing at random, the Markov chain Monte Carlo method will be used to impute missing primary outcomes at T1 separately by each group [20]. Variables used to impute missing values include background characteristics that have strongly predicted diagnosis and treatment competence among primary health care providers in the literature (age, gender, educational level, training background, and years of practice) and baseline values of such outcomes [21,22]. The multiple imputation will be implemented using the “multiple imputation” function embedded in SPSS (version 26.0; IBM Corp). Chi-square and independent-sample 2-tailed *t* tests will be performed to examine the between-group balance of potential confounders at baseline. Multilevel linear regression models (level 1: individual physicians; level 2: township health centers) will be fit to analyze the between-group difference in the primary outcome measured at T1 after adjusting for baseline value of the primary outcome and other significant baseline confounders. Random intercept models will be used to allow the intercept of the regression model to vary across centers, which can account for intracorrelated nested data. SPSS (version 26.0) will be used for data analysis, with 2-sided *P*<.05 considered statistically significant.

## Results

The study was funded in April 2019. The results are expected to be published in December 2027. The study flowchart is shown in Figure 1. Study recruitment started on August 1, 2026. By August 15, 2026, a total of 18 township health centers had been randomly assigned to either the intervention (9 centers) or the control (9 centers) group. A total of 216 physicians from these centers completed baseline assessment; 121 were randomly assigned to the intervention group, and 95 were assigned to the control group. Recruitment will be completed by the end of November 2026. The follow-up assessment at T1 will start in September 2026. Data collection is expected to be concluded in January 2027.

**Figure 1.**
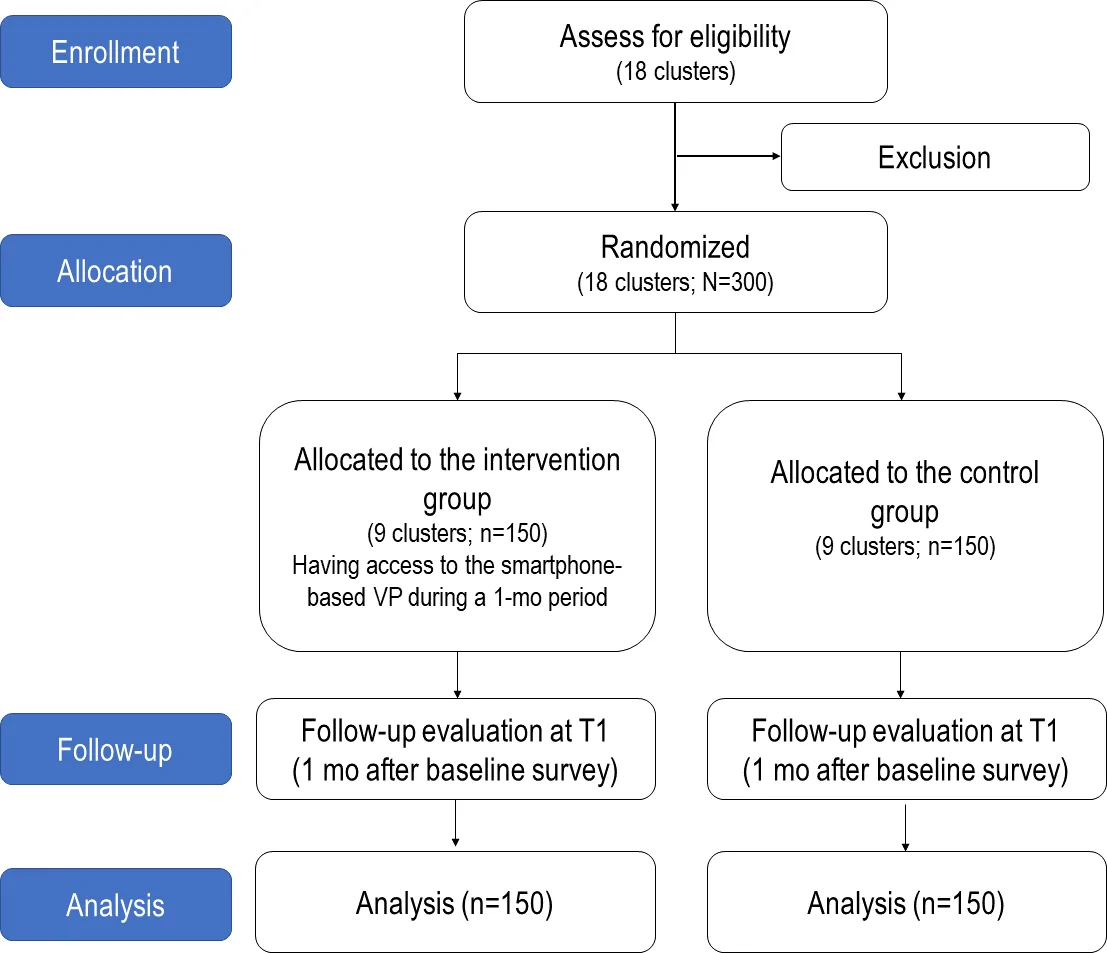
CONSORT (Consolidated Standards of Reporting Trials) flowchart of the cluster randomized controlled trial. VP: virtual patient.

## Discussion

### Anticipated Principal Findings and Comparison to Prior Work

We expect that physicians in the intervention group will have higher scores representing overall diagnosis and treatment competence at T1 compared to those in the control group. Most existing studies evaluating the effectiveness of VPs or smartphone-based VPs have targeted medical students to improve their clinical reasoning skills [13]. There is a dearth of studies applying smartphone-based VPs for other health care providers, including primary health care providers. The findings of our RCT may contribute to the existing literature to address such a knowledge gap. Most of the existing VPs or smartphone-based VPs only cover 1 or 2 specific clinical conditions (eg, surgery or cardiorespiratory conditions) [23-25]. In contrast, our smartphone-based VP covers 11 conditions that could represent common clinical encounters in primary health care settings in rural China, which is more comprehensive and context relevant. In the literature, a number of existing VPs were less interactive and presented the patient history already completed [13]. Ours requires users to interact with the VP to collect patient history, which could better simulate clinical encounters in real-world settings. Similar to previous studies, our RCT will use VP cases to evaluate the primary outcome. Some previous studies have used unannounced standardized patients to evaluate the effectiveness of VPs. Although using unannounced standardized patients is considered the gold standard to evaluate diagnosis and treatment competence, it is less practical in resource-limited settings such as rural China [26,27]. In line with previous studies, our outcome focused on the process (eg, whether the clinical pathway and collection of clinical information were appropriate) in addition to the accuracy of the diagnosis and treatment plans [13].

### Potential Implications of This Study

The underlying purpose of this study is to improve the quality of primary health care services in rural China and, hence, facilitate the achievement of universal health coverage. Existing training methods (eg, traditional face-to-face workshops and unannounced standardized patients) are resource demanding and less practical for resource-limited settings such as rural China [28,29]. In our groundwork, our smartphone-based VP demonstrated high levels of feasibility and acceptability among primary health care physicians in rural China. The VP, if found to be significant and translated into service, will provide a feasible and effective alternative training tool to address service gaps.

### Limitations

This study has some limitations. First, the smartphone-based VP is limited to physicians who have access to smartphones. In 2025 in mainland China, 99% of the counties and villages had access to fourth-generation mobile communication network technology, and 80% of its population had smartphones [30]. In our feasibility study, no physician was excluded due to smartphone inaccessibility. It is expected that very few physicians in rural China do not have access to a smartphone. Second, we are not able to collect information from physicians who refuse to participate in the trial. Physicians who refuse may have different characteristics compared to participants. It is possible that physicians who have a higher motivation to use the VP will be more willing to join the study. There may be selection bias. Third, the follow-up period is short (1 month) due to the funding scope. The limited ability to observe the long-term effects of the smartphone-based VP is a major limitation of this study. Moreover, the participants will be recruited from one county in western China. Caution should be taken when generalizing the findings to other parts of China.

### Dissemination Plan and Future Studies

Findings of this RCT will be disseminated to health authorities (eg, health bureau) and township health centers through workshops and seminars. The findings will also be disseminated through presentations in local and international conferences and journal publications. Once the cluster RCT is completed and the smartphone-based VP has been shown to be superior to the standard of care alone, an implementation study will be undertaken in township health centers in rural China. The objectives of the implementation study will be informing implementation strategies of the smartphone-based VP to evaluate implementation reach and effectiveness and facilitate adoption of this training tool in primary health care settings.

### Conclusions

In summary, this study has several implications. The findings will extend the application of smartphone-based VPs in medical training. If the smartphone-based VP is proven effective in increasing diagnosis and treatment competence among primary health care physicians in rural China, it will provide a feasible and effective alternative training tool to address the existing service gaps.

## Supplementary material

10.2196/106849 Checklist 1 SPIRIT 2025 checklist.

10.2196/106849 Peer Review Report 1 Peer review report by the Special CMB Research Program in Health Policy and Systems Sciences, China Medical Board.

## Abbreviations

DE: design effect
RCT: randomized controlled trial
SPIRIT: Standard Protocol Items: Recommendations for Interventional Trials
VP: virtual patient
