# Supplementary material for: Efficacy of a Smartphone-Based Virtual Patient in Increasing Clinical Competence Among Primary Health Care Providers: Protocol for a Cluster Randomized Controlled Trial
Source: JMIR Res Protoc. 2026 Nov 6;15:e106849. doi: 10.2196/106849 (PMC13742300; doi:10.2196/106849)
Supplement: Supplementary file 2 [file resprot-v15-e106849-s002.pdf]

Special CMB Research Program in Health Policy and Systems Sciences
Proposal Review Result

Feb 12, 2019

**University Abbreviation**	LZU
**PI name**	Wang Xiaohui
**Project title**	Implementation and Evaluation of Smartphone-based Virtual Patients as an Innovative Training Approach among Primary Healthcare Workers in Rural China

Reviewer Comment 1

In an era of rapidly advancing digitalization, and given that technological breakthroughs in virtual patient technology have already been achieved, the application of this model in primary healthcare delivery demonstrates certain innovation and necessity. Since the technology is mature, the proposal should provide more detail (e.g. intended positioning, service content, operating procedures), so as to demonstrate whether it is practically implementable.

The proposal should place greater emphasis on implementation and on the development of evaluation tools. When assessing the efficacy of smartphone-based virtual patient, what specific outcome indicators will be used? The operational feasibility and evaluation components should therefore be further elaborated.

Reviewer Comment 2

The applicant has a strong research foundation. The proposed study, which provides smartphone-based virtual patient to physicians working in rural health centers, addresses a topic with considerable practical value. The research content and objectives are relatively clearly described, and the study plan is detailed and specific.

However, as a proposal submitted to the section on Health Policy and Systems Sciences, the study should articulate a clearly defined scientific question and fully demonstrate its scientific rigor and innovation, both theoretically and practically, rather than merely applying and evaluating an existing smartphone-based virtual patient model. In addition, the characteristics of the smartphone-based virtual patient model alone do not adequately demonstrate the distinctive features or innovative contribution of this project.

Reviewer Comment 3

1. The project uses smartphones and virtual patients to train rural primary healthcare providers and evaluates the training outcomes through intervention and control-group comparisons. This represents a considerable degree of innovation.

2. The study is well designed. The randomized controlled clinical trial is appropriately applied, the research methods are suitable, the sample size calculation is correct, and issues such as medical ethics and informed consent have been taken into consideration.

3. The overall proposed budget appears somewhat high, while insufficient funding seems to have been allocated to the actual research activities, particularly the costs of conducting the randomized controlled clinical trial. Some of the funds allocated to travel and personnel remuneration should be reallocated to direct study-related expenses.

Reviewer Comment 4

The proposed topic focuses on the training of rural primary healthcare providers and explores a new training model based on modern information technology, giving it a certain practical value. The study adopts a matched-pair design, and the overall research approach is relatively clear.

It is recommended that a health economic evaluation be incorporated into the assessment of training efficacy. The costs, suitability, and other relevant aspects of the new training model should be comprehensively evaluated. Such an evaluation would support the further exploration of innovative strategies for the broader implementation and scale-up of the model.

Reviewer Comment 5

This is a randomized controlled trial of smartphone-based virtual patient training. The study pathway is clear, the selected methods are appropriate, and the study protocol is feasible. However, several aspects require further improvement:

1. The proposal does not clearly explain how smartphone-based virtual patient will be delivered to primary healthcare providers. It is unclear whether participants will be brought together at a centralized location to receive group-based instruction or whether they will independently use their smartphones to view virtual patient videos.

2. The proposal should provide information on the preliminary application and pilot testing of the successfully developed mobile virtual patient platform.

3. The study aims to evaluate the efficacy of the smartphone-based virtual patient. However, the proposal lacks a review of the relevant literature concerning smartphone-based virtual patient outcome indicators and assessment tools. In addition, these indicators and tools are not described in sufficient detail in either the research methods or the study content. These elements constitute the foundation of an evaluation study.
